# Association Between Serum Thyroid-Stimulating Hormone Levels and Salivary Microbiome Shifts

**DOI:** 10.3389/fcimb.2021.603291

**Published:** 2021-02-26

**Authors:** Ting Dong, Fen Zhao, Keyong Yuan, Xiaohan Zhu, Ningjian Wang, Fangzhen Xia, Yingli Lu, Zhengwei Huang

**Affiliations:** ^1^ Department of Endodontics, Shanghai Ninth People’s Hospital, College of Stomatology, Shanghai Jiao Tong University, School of Medicine, Shanghai, China; ^2^ National Clinical Research Center for Oral Diseases, Shanghai, China; ^3^ Shanghai Key Laboratory of Stomatology, Shanghai, China; ^4^ Shanghai Research Institute of Stomatology, Shanghai, China; ^5^ Institute and Department of Endocrinology and Metabolism, Shanghai Ninth People’s Hospital, Shanghai Jiao Tong University School of Medicine, Shanghai, China

**Keywords:** thyroid stimulating hormone, salivary microbiome, 16S rDNA sequencing, insulin resistance, community shift

## Abstract

High serum thyroid-stimulating hormone (TSH) levels are linked to many metabolic disorders, but the effects of TSH levels on the oral microbiota are still largely unknown. This study aimed to explore the association between the salivary microbiome in adults and serum TSH levels. Saliva and fasting blood samples were obtained from a health census conducted in Southeast China. All participants were divided according to serum TSH levels. The microbial genetic profiles and changes were acquired by 16S rDNA sequencing and bioinformatics analysis. Relevant anthropometric and biochemical measurements such as insulin resistance, blood lipids, and body composition were evaluated with laboratory tests and physical examinations. The salivary microbiome in individuals with higher TSH level showed significantly higher taxa diversity. Principal coordinates analysis and partial least squares discriminant analysis showed distinct clustering in the Abnormal and Normal Groups (Adonis, *P*=0.0320). *Granulicatella* was identified as a discriminative genus for comparison of the two groups. Fasting serum insulin, Homeostatic Model Assessment for Insulin Resistance, and hemoglobin A1 were elevated in the Abnormal Group (*P*<0.05), showing the presence of insulin resistance in individuals with abnormal higher serum TSH levels. Distance-based redundancy analysis revealed the association of this distinctive difference with salivary microbiome. In conclusion, shifts in microbial profile were observed in the saliva of individuals with different serum TSH levels, and insulin resistance may play an important role in the biochemical and microbial alteration.

## Introduction

The oral microbiome is a collection of microorganisms that colonize the human oral cavity and constitute an important microenvironment (Zhang et al., 2015). The oral microbiome harbors 50–100 billion bacteria in the oral cavity, and it has attracted increased attention by researchers and clinicians for its accessibility compared with microbiomes at other sites (Krishnan et al., 2017). Located at the beginning of the digestive tract, the oral microbiome plays a critical role in regulating nutrient absorption, substance metabolism and immune responses (Gao et al., 2018). The oral microbiome is dependent on the environment, and it is subject to changes in the environment.

Shifts in the oral microbiome are observed in some diseases or when the environment changes. Extensive studies have shown major changes in bacteria in most oral diseases, such as dental caries and periodontitis. Changes are also commonly seen in various systemic disorders, such as gastrointestinal, nervous, endocrine, immune, and cardiovascular diseases (Si et al., 2017). The oral microbiome is also related to various forms of metabolic dysbiosis, such as diabetes, hyperglycemia, and obesity (Saeb et al., 2019; De Andrade et al., 2020; Wei et al., 2020). Diabetes is reported to cause a shift in the oral microbiome with 16S rRNA sequencing and the reason for this shift may be enhanced expression of interleukin-17 in diabetes (Xiao et al., 2017). Our previous work has detected decreased richness and diversity of the oral microbiome during and after radiotherapy (Gao et al., 2015), and major shifts in supragingival microbiota during pregnancy (Lin et al., 2018).

Thyroid-stimulating hormone (TSH) is secreted by the adenohypophysis. TSH plays a critical regulatory role in the maintenance of normal thyroid function and is regulated by the hypothalamic–adenohypophysis–thyroid axis (Kopp, 2001). Thyroid hormone (TH) plays a significant role in maintaining metabolic homeostasis throughout life and is involved in the regulation of body composition, lipid metabolism, and insulin resistance (Asvold et al., 2013; Mullur et al., 2014; Martinez and Ortiz, 2017). The proportion of individuals with increased TSH level in the general population has increased in recent years. Hypothyroidism, subclinical hypothyroidism and thyroid hormone resistance syndrome are becoming common diseases in the general population (Lefevre, 2015). Elevated serum TSH level may cause organ damage and metabolic disorder (Ruhla et al., 2010). Various changes in physiological indicators and metabolic levels are associated with high TSH levels. Lipid metabolism [including total cholesterol (TC), triglyceride (TG), and low-density lipoprotein cholesterol (LDL-C)] deteriorates with increasing TSH level (Teixeira Pde et al., 2008; Ren et al., 2019). High TSH level is also associated with other cardiovascular risk factors, such as alteration in blood pressure and increased atherosclerosis (Delitala et al., 2017; Ren et al., 2019). Increasing TSH level is linked with insulin resistance and type 2 diabetes (Bos et al., 2017; Jun et al., 2017; Laclaustra et al., 2019).

The oral microbiome is affected by physiological processes and changes in the environment, while the microenvironment and various metabolic processes are disturbed by increased levels of TSH, which is assumed to cause shifts in the oral microbiome. However, there is limited information on the correlation between changes in the oral microbiome and levels of serum TSH. In this study, saliva samples were obtained from volunteers who were divided into Normal and Abnormal Groups according to their serum TSH levels. Illumina MiSeq PE300 sequencing and bioinformatics analysis were used to determine the shifts in salivary microbial profiles with different serum TSH levels. These findings may provide a deeper understanding of the oral ecological dysbiosis associated with high TSH levels.

## Materials and Methods

### Participant Recruitment

Participants aged 45–60 years were recruited from a health census conducted in Southwest China. The inclusion criteria were: (1) Han race; (2) residence in the same community for >5 years; (3) normal measured THs; and (4) no iodine contrast agent examination or amiodarone intake in the past 3 months. The exclusion criteria were: (1) type 1 or 2 diabetes mellitus; (2) symptomatic liver and cardiovascular diseases; (3) cancer or cancer history; (4) other chronic inflammatory disease; (5) oral diseases: untreated oral abscess, precancerous lesions, and oral cancer, oral fungal infection, missing more than eight teeth, etc.; (6) long-term heavy smoking and alcohol consumption; (7) taking antibiotics for >5 days in the last 6 months; and (8) pregnant or lactating women. This study followed the Declaration of Helsinki on medical protocols and ethics and obtained approval from the regional Ethical Review Board of Shanghai 9th Hospital affiliated to Shanghai Jiaotong University, School of Medicine (2019-T295-1). Written informed consent was obtained from all participants.

### Anthropometric and Biochemical Measurements

All of the participants were required to fast overnight before physical examination. The anthropometric parameters included height, body mass index (BMI), waist circumference (WC), hip circumference (HC), and blood pressure. Questionnaires and fasting blood samples were collected for demographic information and biochemical measurements, respectively. The clinical variables included: TSH, triiodothyronine (T3), tetraiodothyronine (T4), TC, TG, LDL-C, high-density lipoprotein cholesterol (HDL-C), uric acid (UA), fasting blood glucose (FBG), fasting serum insulin (FSI), and hemoglobin A1C (HbA1C). Homeostatic Model Assessment for Insulin Resistance (HOMA-IR) was calculated according to the formula: HOMA-IR = FBG (mmol/L) × FSI (mU/L)/22.5 (Bos et al., 2017).

### Group Definition

We recruited 114 participants. Those with serum TSH level >4.2 mIU/L were defined as the Abnormal Group (n=20, 4.20–9.22 mIU/L). The Normal Group (n=20, 0.6–4.20 mIU/L) was screened from participants with serum TSH level no more than 4.2 mIU/L, using MedCalc Statistical Software version 19.0.7 (MedCalc Software, Ostend, Belgium) with case–control matching. The variables were set to an exact match between the sexes and a maximum allowable age difference of 3. All participants included possessed normal T3 and T4. The reference ranges were 0.27–4.20 mIU/L for TSH, 1.30–3.10 nmol/L for T3, and 66.0–181.0 nmol/L for T4 as provided by the laboratory.

### Saliva Sample Collection

Unstimulated saliva samples were collected between 08:00 and 10:00 h before eating in the morning, as described in the Manual of Procedures for the Human Microbiome Project (https://www.hmpdacc.org/hmp/doc/HMP_MOP_Version12_0_072910.pdf). Participants were asked to refrain from drinking, smoking, or oral hygiene procedures for at least 1 h before saliva collection. Saliva samples were collected in test tubes and stored in liquid nitrogen at minus 196 degrees immediately for 5 days until sequencing.

### DNA Extraction, Amplification, and High-Throughput Sequences

Total bacterial DNA was extracted using the QIAamp DNA Mini Kit (Qiagen, Valencia, CA, USA) and then quantified using NanoDrop 2000 UV-vis spectrophotometer (Thermo Scientific, Wilmington, DE, USA) and 1% agarose gel electrophoresis. The V3–V4 region of 16S rRNA genes was amplified using the following primers: 338F forward primer (5′-ACTCCTACGGGAGGCAGCAG-3′) and 806R reverse primer (5′-GGACTACHVGGGTWTCTAAT-3′) by thermocycler PCR system (GeneAmp 9700; Applied Biosystems, Carlsbad, CA, USA) as described previously (Lin et al., 2018). The purified amplicons were sequenced using the Illumina Miseq PE300 platform (Illumina, San Diego, CA, USA) according to the standard protocol.

### Data Processing and Bioinformatics Analyses

All anthropometric and biochemical comparisons were made between the Abnormal and Normal Groups using unpaired *t*-test, except for “Gender”, which was analyzed by χ^2^ test. Operational taxonomic units (OTUs) were clustered with 97% similarity using UPARSE (version 7.1, http://drive5.com/uparse/) and matched to a database (SILVA 106; https://www.arb-silva.de/) for taxonomic analysis. A modified OTUs table was generated for subsequent analysis after subsampling according to the minimum number of sample sequence. Alpha diversity indexes (Ace, Chao, Shannon and Simpson) and co-occurrence networks of the 50 most abundant genera of each group were calculated to evaluate the diversity and construction of community species using MOTHUR (version 1.30.2, https://www.mothur.org/wiki/Download_mothur). Community barplot analysis and heatmaps on genus level were created *via* R platform (version 3.6.1) to describe the species composition in different groups. The weighted UniFrac principal coordinates analysis (PCoA) (Lozupone et al., 2007) and partial least squares discriminant analysis (PLS-DA) (Lee et al., 2018) were performed to evaluate the variances of sample community composition using QIIME. The linear discriminant analysis (LDA) effect size analysis (LEfSe, http://huttenhower.sph.harvard.edu/galaxy) was applied to identify the most discriminatory taxa among groups from phylum to genus level with logarithmic LDA score >2.0 regarded as discriminative species (Segata et al., 2011). The unweighted Unifrac distance-based redundancy analysis (db-RDA) and Spearman’s rank correlation coefficient were operated to quantitatively evaluate the multicollinearity relationship between environmental/clinical factors and sample species composition (Roberts, 2009). Phylogenetic Investigation of Communities by Reconstruction of Unobserved States (PICRUSt2, version 1.1.0, http://picrust.github.io/picrust/) analysis and Wilcoxon rank sum test with a Benjamini–Hochberg false discovery rate (FDR) correction to adjust *P* values for multiple testing were performed to predict and compare the abundance of Kyoto Encyclopedia of Genes and Genomes (KEGG) pathways in different groups.

### Data Access

All the raw sequences were deposited in the NCBI Sequence Read Archive database. The accession number is SRP273570.

## Results

### Participant Characteristics

There were no differences in sex and age between the Abnormal and Normal Groups, based on case–control matching. The anthropometric and biochemical variables of the two groups were summarized in **Table 1**. FSI, HOMA-IR, and HbA1C were significantly elevated in the Abnormal Group (*P*<0.05), demonstrating the presence of insulin resistance in participants with abnormally higher serum TSH levels. TG, which reflected lipid metabolism status and was affected by thyroid function, was significantly higher in the Abnormal compared with Normal Group (*P*<0.05) and TC was lower in the Abnormal Group.

**Table 1:** Anthropometric and biochemical variables of the participants with normal/abnormal serum thyroid-stimulating hormone (TSH) levels.

Variables	Group Abnormal (n=20)	Group Normal (n=20)	P-value
Age (years)	52.75 ± 5.14	52.95 ± 4.67	0.8982
Gender (M: F) TSH T3 T4 TPOAb	8 : 12 5.55 ± 1.60 1.79 ± 0.35 111.00 ± 17.03 16.79 ± 6.82	8 : 12 2.36 ± 0.68 1.78 ± 0.23 114.30 ± 15.85 29.77 ± 49.99	>0.9999 <0.0001** 0.9662 0.5369 0.2571
TC (mmol/L)	5.15 ± 0.88	5.73 ± 0.91	0.0256*
TG (mmol/L)	2.22 ± 1.48	1.57 ± 0.72	0.0373*
LDL-C (mmol/L)	3.04± 0.57	3.35± 0.44	0.0666
HDL-C (mmol/L)	1.15 ± 0.33	1.42± 0.24	0.0058
FPG (mmol/L)	5.15 ± 0.42	5.00+0.47	0.3066
FSI (mU/L)	5.31 ± 2.36	3.76 ± 2.35	0.0447*
HOMA-IR HbA1c UA	1.22 ± 0.58 5.52 ± 0.27 317.10 ± 60.94	0.83 ± 0.53 5.24 ± 0.40 312.50 ± 67.31	0.0322* 0.0131* 0.8201
Height (cm)	161.70 ± 6.88	160.40 ± 7.63	0.5733
BMI NC (cm) WC (cm) HC (cm)	23.80 ± 2.77 35.59 ± 4.01 83.90 ± 7.06 96.36 ± 6.91	24.04 ± 2.82 36.00 ± 3.30 81.49 ± 9.29 96.34 ± 4.20	0.7884 0.7227 0.3625 0.989
SBP (mm Hg)	136.30 ± 15.10	127.60 ± 18.74	0.1162
DBP (mm Hg)	82.05 ± 10.34	78.30 ± 14.10	0.3436

Data are mean ± standard deviation. *P<0.05, **P<0.0001. Anthropometric and biochemical variables was analyzed using unpaired t-test except for “Gender”, which was analyzed with χ^2^ test. DBP, diastolic blood pressure; FBG, fasting plasma glucose; SBP, systolic blood pressure; TPOAb, thyroid peroxidase antibody.

### Community Structure and Species Composition of Oral Microbiome

**Figure 1:**
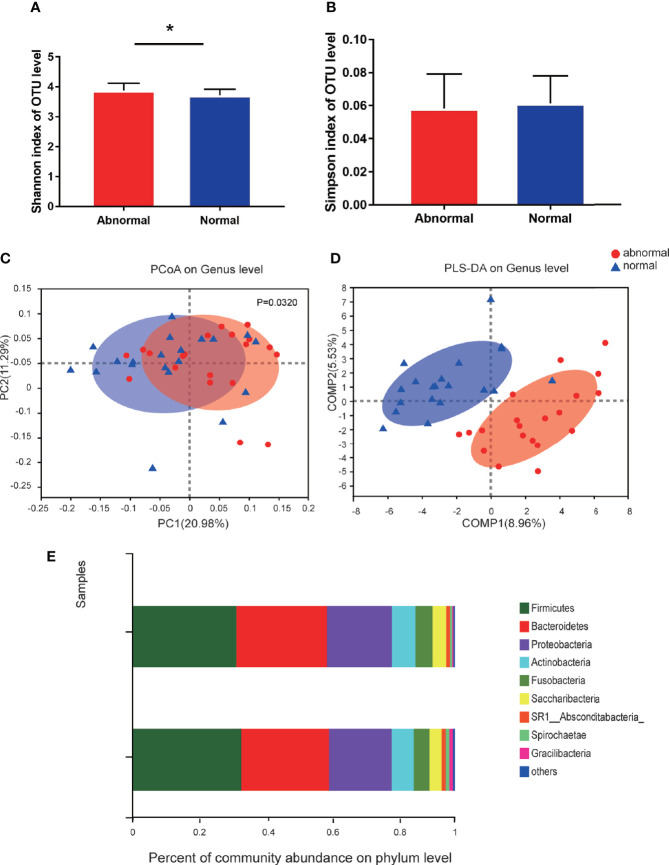
Diversity and species composition of salivary microbiome in the Abnormal and Normal Groups. **(A)** Chao Index of operational taxonomic unit (OTU) level, *P<0.05. **(B)** Simpson index of OTU level. **(C)** Weighted UniFrac PCoA of genus level, *P*<0.05. **(D)** partial least squares discriminant analysis (PLS-DA) of genus level showed more obvious separation in the horizontal coordinate axis. **(E)** Community bar plot analysis showing the top 10 phyla with highest abundance.

**Figure 2:**
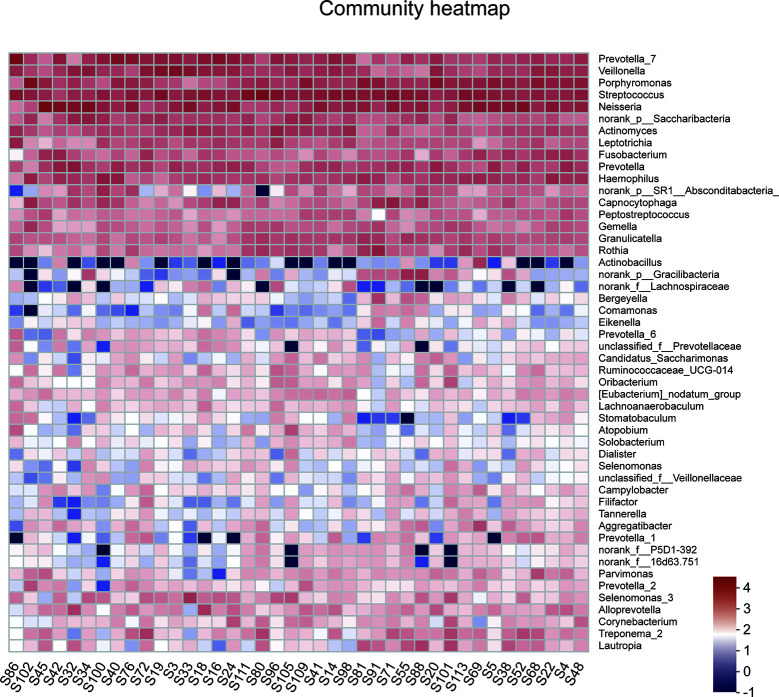
A heatmap of the abundance of top 50 abundant genera in each sample. The right side of the legend shows the color range of different R values. The color gradient of the color block was used to show the variation in abundance of different species in the sample.

**Figure 3:**
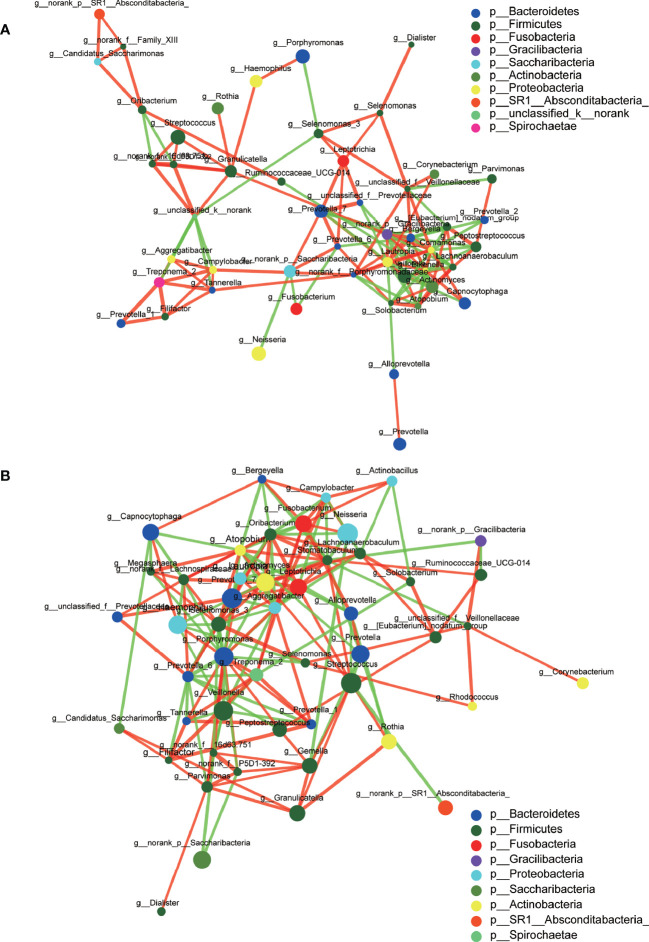
Co-occurrence networks of the top 50 abundant genera in the Abnormal **(A)** and Normal **(B)** Groups. The size of the node indicates the mean relative abundance of the corresponding genus. The same color represents the genera belonging to the same phylum. The thickness of connecting lines corresponds to the coefficient values. The red and green lines indicate a positive and negative correlation, respectively.

A total of 1,216,520 high-quality sequences were produced, and 21 phyla, 38 classes, 71 orders, 119 families, 258 genera, 514 species, and 978 OTUs were identified for the Abnormal and Normal Groups. The microbial community from the Abnormal Group indicated significantly greater community richness than the Normal Group (*P*<0.05, **Figure 1A**), but a smaller Simpson Index at the OUT level (**Figure 1B**). The samples from the Abnormal and Normal Groups shared a similar core microbiome (Gao et al., 2015). The top phyla of both groups (including *Firmicutes*, *Bacteroidetes*, *Proteobacteria*, *Actinobacteria*, *Fusobacteria*, *Saccharibacteria*, *SR1 Absconditabateria*, *Spirochaetes*, and *Gracilibacteria*) accounted for >99% of the total phyla found in the salivary samples of the Abnormal and Normal Groups and exhibited analogous community species composition (**Figure 1E**). The top 50 abundant genera in each sample are displayed in the heatmap (**Figure 2**). There was an obvious tendency for separation in the PC1 axis in PCoA and COMP 1 axis in PLS-DA, indicating the differences in sample community composition in the Abnormal and Normal Groups (**Figures 1C, D**). The co-occurrence network of the top 50 abundant genera in the salivary microbiome exhibited slightly different community structures, but both mainly consisted of *Bacterioidetes* and *Firmicutes* (**Figure 3**).

### Alterations of the Salivary Microbial Phylotypes Associated With Serum TSH Levels

**Figure 4:**
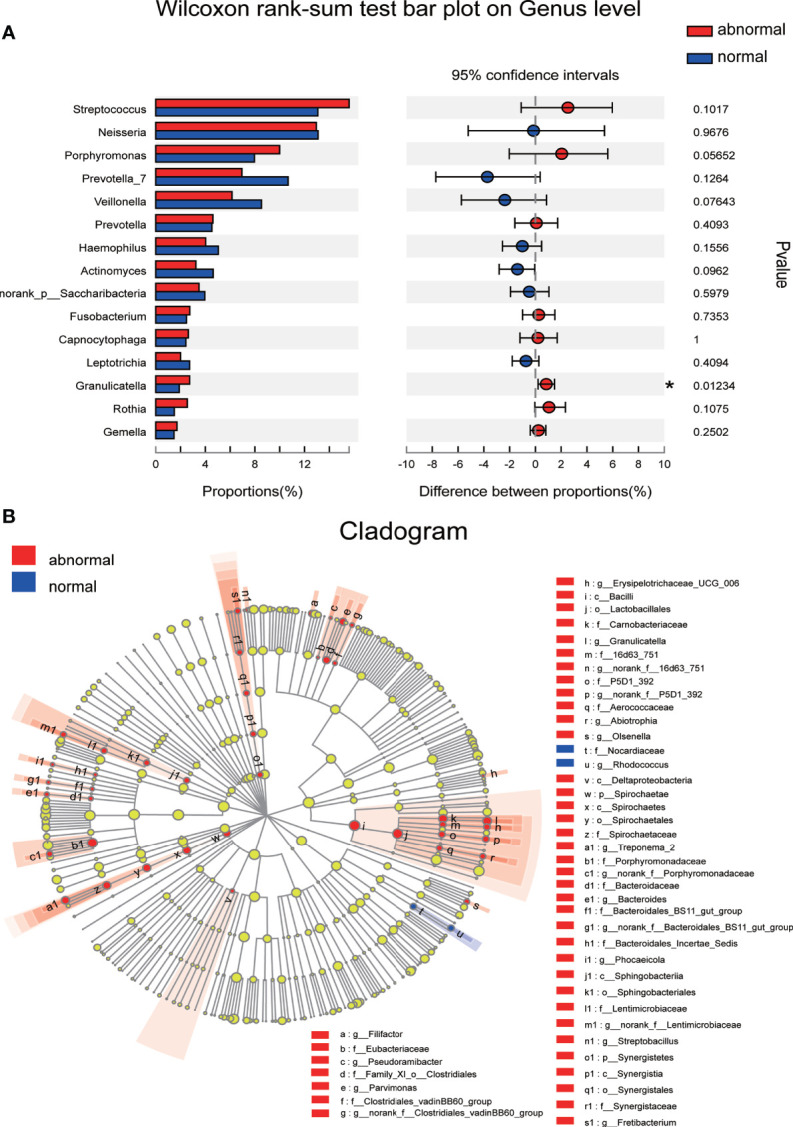
Discriminative species with maximum abundance difference in the Abnormal and Normal Groups. **(A)** Discriminative species at genus level were identified using Wilcoxon rank-sum test, *P < 0.05. **(B)** A cladogram for taxonomic representation based on LEfSe. Red indicates enrichment in samples from the Abnormal Group, and blue indicates the taxa enriched in samples from the Normal Group (LDA>2.0, *P*<0.05).

The significance level of the difference in species abundance was evaluated with Wilcoxon rank-sum test according to the obtained community abundance data, and species with significant variances between groups were obtained at the genus level. *Granulicatella* was found to possess significantly higher abundance in the Abnormal Group compared with the Normal Group (**Figure 4A**). The most discriminatory taxa between groups from phylum to genus level were further identified using LEFSe with logarithmic LDA score >2.0. Forty-five taxa showed differential distribution in the two groups. *Lactobacillales* at the order level, *Porphyromonadaceae*, *Carnobacteriaceae*, and *Spirochaetae* at the family level, and *Granulicatella*, *Treponema*, and *Streptobacillus* at the genus level were more prevalent in the Abnormal Group, while *Rhodococus* and *Nocardiaceae* were more abundant in the Normal Group (**Figure 4B**).

### Associations Between Clinical Variables and Salivary Microbiome

**Figure 5:**
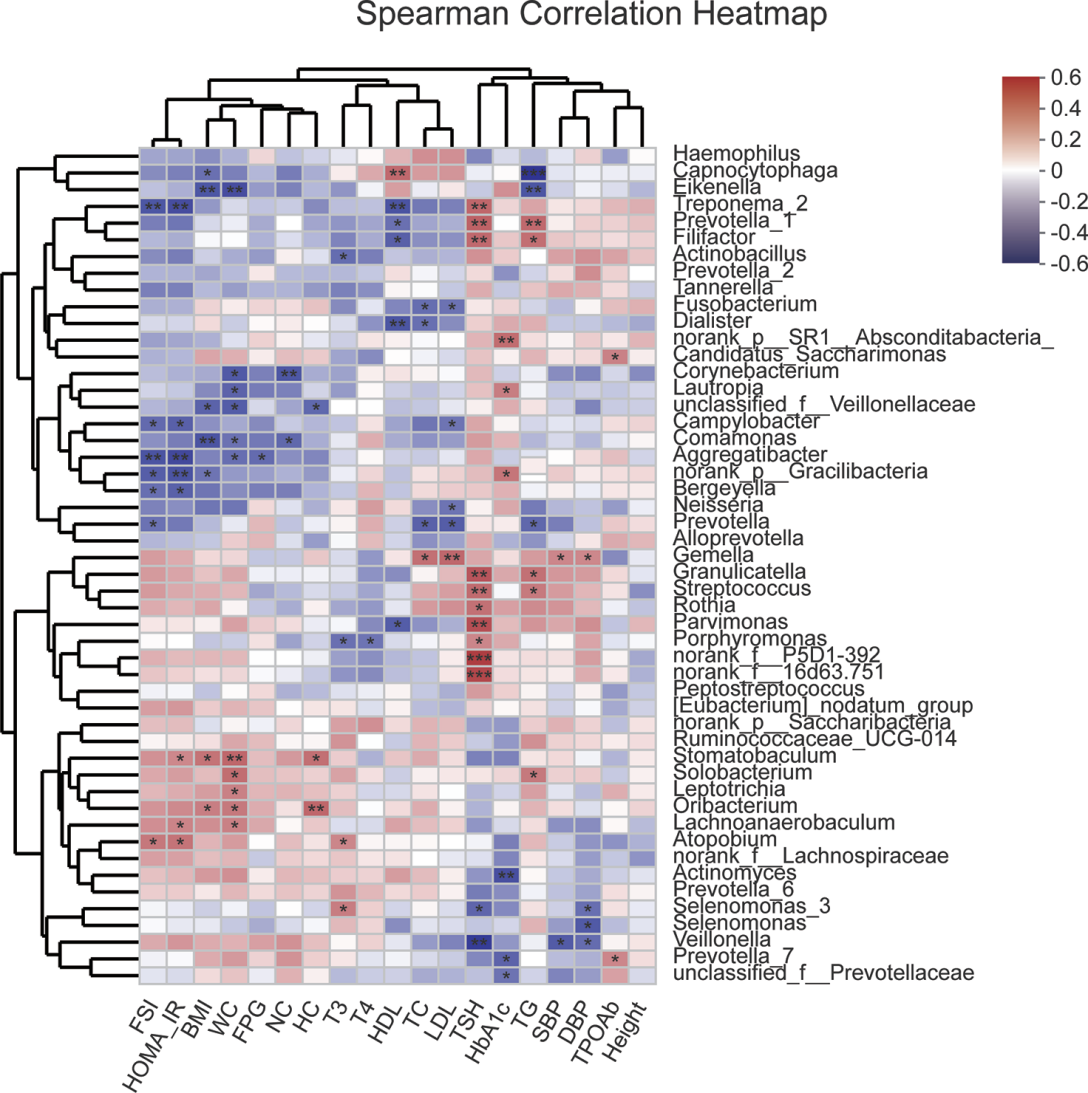
Heatmap of Spearman correlation analysis between the top 50 abundant salivary microbiota and clinical variables. R value shows in different colors, red indicates positive correlation while blue indicates negative correlation. The darker the color, the greater the correlation coefficient. Species clustering trees were presented in the left side of the heat map. *P<0.05; **P<0.01; ***P<0.001.

Unweighted Unifrac db-RDA was performed to analyze the relation between the microbial community and clinical variables. The degree of explanation of obesity-related [BMI, neck circumference (NC), WC and HC] and glucose-metabolism-related (HOMA-IR, HbA1C, and FSI) variables occupied the main part (**Figure S2**). HOMA-IR contributed most to the degree of explanation with r^2 =^ 0.3165, which had significance, followed by FSI (r^2 =^ 0.2844), BMI (r^2 =^ 0.2282), WC (r^2 =^ 0.1889), NC (r^2 =^ 0.1589), and HC (r^2 =^ 0.1538). Spearman correlation coefficient of the top 50 abundant genera and clinical variables was visualized using a heatmap (**Figure 5**). R value is shown in different colors; red indicates positive correlation and blue negative correlation.

### Alterative Predictive Metagenome Functional Profiling Along With Serum TSH Levels

The functional profile of the salivary microbiome along with changes in serum TSH levels was further explored using PICRUSt 2 based on high-quality amplicon sequencing. Wilcoxon rank sum test with a Benjamini–Hochberg FDR correction to adjust *P* values for multiple testing was used for detection. Carbohydrate metabolism accounted for the highest proportion of the functional composition of the microbial community in the saliva samples at KEGG level 2 (**Figure S3A**). The top 10 functional components of abundance were mainly concerned with carbohydrate, nucleotide, amino acid and energy metabolism and material transport at KEGG levels 2 and 3 (**Figure S3A, B**), but no significant difference was found between the two groups after multiple testing.

## Discussion

The salivary microbiome comprises a mixture of microorganisms from all oral sites and is more accessible than the microbiome of other sites, which makes saliva an easy and non-invasive alternative to other sampling options (Belstrøm, 2020). The salivary microbiome is shaped by the surrounding environment to some extent, and any fluctuation in that environment may cause variation in the microbiome. Shifts in the salivary microbiome have been reported in many oral and non-oral diseases (Said et al., 2014; Wu et al., 2018; Yang J. et al., 2018). Disturbances in hormone and metabolic levels often lead to changes or even dysbiosis in the oral microbiome. Sex hormones have been confirmed to exert a critical influence on the oral microbiome, and fluctuations in sex hormones can change the composition and structure of oral microbiota, with puberty and pregnancy being most typical (Kumar, 2013). In our previous study that focused on the shifts in oral microbiota associated with pregnancy, 53 OTUs were observed to have positive correlations with sex hormones, especially *Prevotella* spp. and *Treponema* spp. (Lin et al., 2018). Several studies have reported an association of the salivary microbiome with diabetes and detected decreased bacterial diversity in patients with diabetes (Sabharwal et al., 2019; Saeb et al., 2019).

An elevated serum TSH level may cause various changes in physiological indicators and metabolic levels (Mullur et al., 2014; Martinez and Ortiz, 2017). Therefore, it is easy to imagine that the salivary microbiome changes with increasing TSH levels and accompanying physiological changes. Elevated microbial abundance reflected by greater Ace and Chao Index was observed in the Abnormal Group, which may indicate changes in the microenvironment and the original microbiota. Microbial diversity is generally considered to be tightly associated with the health status of the host. Increased diversity of oral microbiota is often reported in individuals with poor oral conditions (caries, periodontitis, or mucosal diseases) (Pozhitkov et al., 2015; Takeshita et al., 2016) and several metabolic diseases (Si et al., 2017; De Andrade et al., 2020).

The discrepancy in the community structure of the salivary microbiome between the Abnormal and Normal Groups indicated greater microbial shifts once the serum TSH levels exceed the upper limit of normal. *Granulicatella* was distinguished as the discriminative genus between the groups using LEfSe and Wilcoxon rank-sum test. *Granulicatella* is normally associated with health (Sanz et al., 2017). It is considered as a suspicious periodontal disease pathogen, and 16S sequencing technology has confirmed its high detection rate in periodontitis (Lourenço et al., 2014). Decreased abundance of *Granulicatella* has been linked with asthma (Wang et al., 2018), and significantly greater abundance can be detected in many systemic disorders such as obesity (Wu et al., 2018), infective endocarditis (Téllez et al., 2018) and pancreatic cancer (Gaiser et al., 2019). A comprehensive analysis of the oral microbiome found that *Granulicatella* and *Neisseria* were highly enriched in patients with metabolic syndrome (Si et al., 2017). *Porphyromonas*, *Parvimonas*, and *Streptococcus* are also significantly correlated with abnormal TSH levels. *Porphyromonas* is a key pathogen in periodontitis and is closely related to many systemic diseases. A number of studies have explored the role of *Porphyromonas* in atherosclerosis (Giles et al., 2020), Alzheimer’s disease (Miller and Scott, 2020), rheumatoid arthritis (Cheng et al., 2020), diabetes (Minty et al., 2019), and adverse pregnancy outcomes (Chopra et al., 2020) and nonalcoholic fatty liver disease (Clos-Garcia et al., 2020; Wang et al., 2020; Zhou et al., 2020). *Parvimonas* have been reported to be a potential carcinogenic pathogen, which is positively associated with oral squamous cell carcinoma (OSCC) (Zhou et al., 2020), colorectal cancer (Clos-Garcia et al., 2020), and gastric cancer (Gantuya et al., 2020). *Streptococcus* is one of the most common bacteria in the oral cavity, and is predominant in the saliva of patients with liver diseases (Li et al., 2020) and is associated with OSCC (Yang C. Y. et al., 2018). A significantly higher relative abundance of *Streptococcus* has been observed in type 1 diabetes (Leiva-Gea et al., 2018) and nonalcoholic fatty liver disease (Ponziani et al., 2019).

We used co-occurrence networks of the Top 50 abundant genera to predict inter-genera correlations of saliva in the two groups, which exhibited different community structures, but both mainly consisted of *Bacterioidetes* and *Firmicutes*. The degree of interconnectedness among the genera in the Normal Group was higher than that in the Abnormal Group. This indicates that the increase in TSH level was related to the decrease in species richness, as well as the decrease in the interaction among these bacteria. In contrast, the genera within the Abnormal Group formed smaller clusters with fewer interconnections, suggesting an unknown antagonistic or mutually exclusive relationship, which is in accordance with previous research (Qiao et al., 2018).

TH is necessary for normal development as well as regulating metabolism in humans. It modulates hepatic insulin sensitivity, which is especially important for the suppression of hepatic gluconeogenesis (Mullur et al., 2014). Thyroid disease and diabetes mellitus are the two major endocrine disorders that are treated concurrently nowadays (Waring et al., 2012), so it is not surprising that TH and insulin signaling are related to each other. In the present study, several biochemical variables were demonstrated to differ significantly once serum TSH levels were beyond the normal range, which were mainly clinical indicators of glucose metabolism (including FSI, HOMA-IR, and HbA1C) and lipid metabolism (TC and TG). Insulin resistance is a metabolic disorder that affects many insulin-regulated pathways, and it is characterized by reduced action of insulin, usually described with HOMA-IR (Artunc et al., 2016). Several studies have reported that elevation in TSH levels is correlated with insulin resistance (Vyakaranam et al., 2014; Ujwal Upadya et al., 2015). Our study suggested the presence of insulin resistance in individuals with abnormally higher serum TSH levels, which is consistent with previous reports (Kouidhi et al., 2013; Vyakaranam et al., 2014; Ujwal Upadya et al., 2015). Higher TG and lower HDL-C were detected in the Abnormal Group, supporting the presence of insulin resistance with comparatively higher TG/HDL-C ratio (Uruska et al., 2018).

db-RDA also indicated that HOMA-IR contributed most to the main explanatory variables affecting distribution of the salivary microbiome. Insulin resistance means that the normal insulin concentration is not sufficient to produce the desired response to its target tissues. In this case, the pancreatic β cells secrete more insulin to overcome hyperglycemia (Onyango, 2018). Insulin exerts its physiological function by binding to tyrosine kinase receptors. Two main parallel pathways are activated after insulin binding: phosphatidylinositol-3 kinase and mitogen-activated protein kinase pathways, while the balance between these two pathways is disrupted under insulin resistance (Kim et al., 2006), which may have an effect on various metabolic processes. Although no significant differences were detected between our two groups with different TSH levels, carbohydrate metabolism was confirmed to have the highest proportion of the functional composition of the microbial community in the saliva samples. This may explain why HOMA-IR contributed most to the main explanatory variables affecting distribution of the salivary microbiome. Lower community diversity was observed in participants with higher TSH levels, which coincides with the suggestion that greater gene richness implies better health (Zhao et al., 2018). Decreased gene functional abundance in the salivary microbiome of individuals with abnormally higher TSH levels may result in decreased biosynthesis of essential amino acids, nucleotides, vitamins, and utilization of carbohydrate and energy, which may be involved in the tendency of insulin resistance. Considering that 16S rDNA sequencing does not yield robust metabolic and functional pathways, several significant variances may be detected using further metagenomic sequencing.

Insulin resistance with abnormally higher serum TSH levels is accompanied with shifts in the salivary microbiome. *Aggregatibacter*, *Treponema 2*, *norank p Gracilibacteria*, *Campylobacter*, and *Bergeyella* were negatively associated with HOMA-IR, while *Atopobium*, *Lachnoanaerobaculum*, and *Stomatobaculum* were positively correlated with HOMA-IR. *Aggregatibacter* is prevalent at multiple oral sites and closely related to aggressive periodontitis (Åberg et al., 2015), and is associated with infective endocarditis (Nørskov-Lauritsen, 2014) and non-alcoholic fatty liver disease (Komazaki et al., 2017). *Treponema denticola* and *Aggregatibacter actinomycetemcomitans* are increased in healthy periodontal sites in patients with insulin-dependent diabetes mellitus compared with those without diabetes mellitus (Aemaimanan et al., 2013). Decreased abundance of *Treponema 2* was recorded after treatment in type 2 diabetic Goto–Kakizaki rats (Zhang et al., 2019). *Campylobacter* is a Gram-negative bacillus and involved in many human diseases including gastroenteritis, abortion, septicemia, inflammatory bowel disease, and periodontitis (Lee et al., 2016). *Campylobacter* has a positive association with higher blood glucose levels, and likely contributes to the development of hyperglycemia (Wei et al., 2020). *Bergeyella* is correlated with volatile sulfur compounds among periodontally healthy adults (Ye et al., 2019), preterm birth (Han et al., 2006), and infective endocarditis (Mulliken et al., 2019). *Atopobium* is correlated with sarcoidosis (Zimmermann et al., 2017) and significantly increased in multiple polypoid adenomas and/or intramucosal carcinomas (Yachida et al., 2019). *Atopobium* and *Stomatobaculum* can also predicted greater fasting plasma glucose (FPG) change and insulin resistance according to a study among 230 diabetes-free adults (Demmer et al., 2019). *Lachnoanaerobaculum* can be isolated from gingivitis lesions (Lim et al., 2019) and is associated with recurrent aphthous stomatitis (Stehlikova et al., 2019). Chronic systemic inflammation has also been linked to increased risk of insulin resistance and the oral microbiome. Inflammation may cause oral dysbiosis and risk of diabetes (Demmer et al., 2017).

There were some limitations to our study. First, although 114 participants were recruited, only 40 were matched in two groups and underwent relevant bioinformatic analysis. A larger study needs to be carried out to verify our preliminary findings and the biological mechanisms involved require further experimental study. To further explore the variance in the genetics and function of the microbiome, metagenomics sequencing may be needed in our next study.

## Conclusions

The salivary microbial profile shifted with serum TSH levels, which was reflected in the increase in taxa diversity and change of community structure and species composition, along with elevated TSH levels. *Granulicatella* was significantly more prevalent in individuals with abnormal TSH levels. Insulin resistance may play an important role in the accompanying biochemical and microbial alterations. These finding provide a new perspective to explore the association of oral ecological dysbiosis with high TSH levels and may contribute to the diagnosis and interventional treatment of hypothyroidism.

## Data Availability Statement

The datasets presented in this study can be found in online repositories. The names of the repository/repositories and accession number(s) can be found below: https://www.ncbi.nlm.nih.gov/, SRP273570.

## Ethics Statement

This study followed the Declaration of Helsinki on medical protocol and ethics and obtained the approval from the regional Ethical Review Board of Shanghai 9th Hospital affiliated to Shanghai Jiaotong University, School of Medicine (2019-T295-1). The patients/participants provided their written informed consent to participate in this study.

## Author Contributions

DT and ZF performed the study design, data analysis, drafting and revising of the work. YKY and ZXH performed the bioinformatics analysis and sampling collection. WNJ, XFZ and LYL provided the relative clinical assessment. LYL and HZW contributed to the study design, clinical sample collection, data analysis, drafting, revising, final approval. All authors contributed to the article and approved the submitted version.

## Funding

This work was supported by grants from the National Natural Science Foundation of China (82071104/81570964/81371143), the Shanghai Clinical Research Center for Oral Diseases (19MC1910600), and partly supported by the Shanghai Ninth People’s Hospital affiliated with Shanghai Jiao Tong University, School of Medicine (JYJC201806/JYLJ201908).

## Conflict of Interest

The authors declare that the research was conducted in the absence of any commercial or financial relationships that could be construed as a potential conflict of interest.

## Supplementary Material

Supplementary Figure 1 Rarefaction curve on the OTUs level. The curve tends to be flat, indicating that the sequencing data volume is reasonable.

Supplementary Figure 2 Unweighted Unifrac db-RDA reflected the relationship between salivary microbiome and clinical variables. Red indicates samples from the Abnormal Group and blue indicates samples from the Normal Group. The red arrow indicates the quantitative clinical variables. The length of the arrow represents the degree of influence (interpretation) of the clinical variables on the species data; the angle between the arrows represents positive and negative correlation (acute angle: positive correlation; obtuse angle: negative correlation; right angle: no correlation).

Supplementary Figure 3 Alterative predictive metagenome functional profiling along with serum TSH levels. **(A)** Relative abundance of predictive metagenome functional profiling of the top 10 abundant KEGG level 2 pathways (pathways with *P*(FDR)<0.05 are shown). **(B)** Relative abundance of predictive metagenome functional profiling of the top 10 abundant KEGG level 3 pathways (pathways with *P*(FDR)<0.05 are shown).
