# Supplementary figures and images for: Association Between Serum Thyroid-Stimulating Hormone Levels and Salivary Microbiome Shifts
Source: Front Cell Infect Microbiol. 2021 Feb 26;11:603291. doi: 10.3389/fcimb.2021.603291 (PMC7952758; doi:10.3389/fcimb.2021.603291)

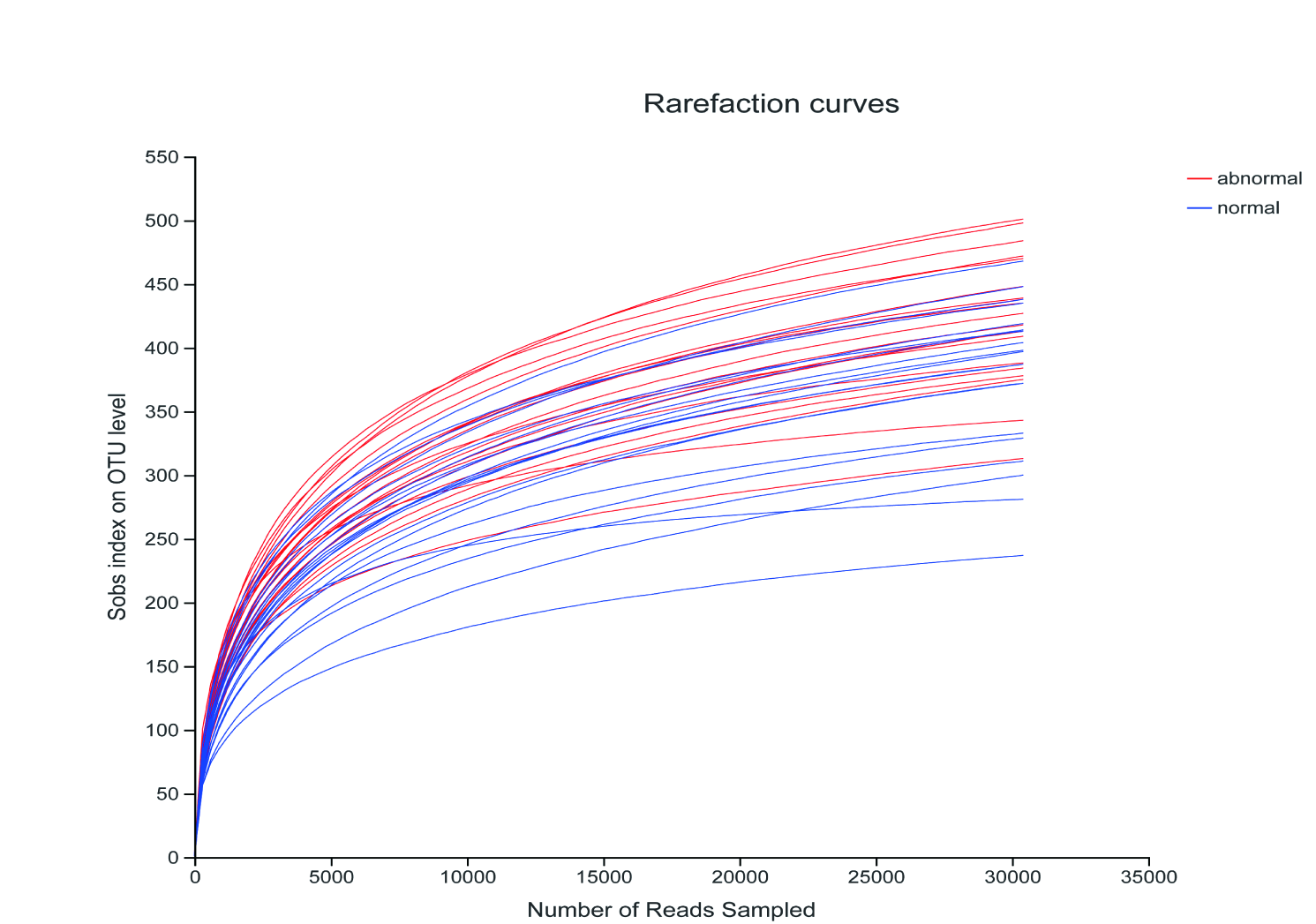

Supplement: Supplementary Figure 1 — Rarefaction curve on the OTUs level. The curve tends to be flat, indicating that the sequencing data volume is reasonable. [file Image_1.tif]

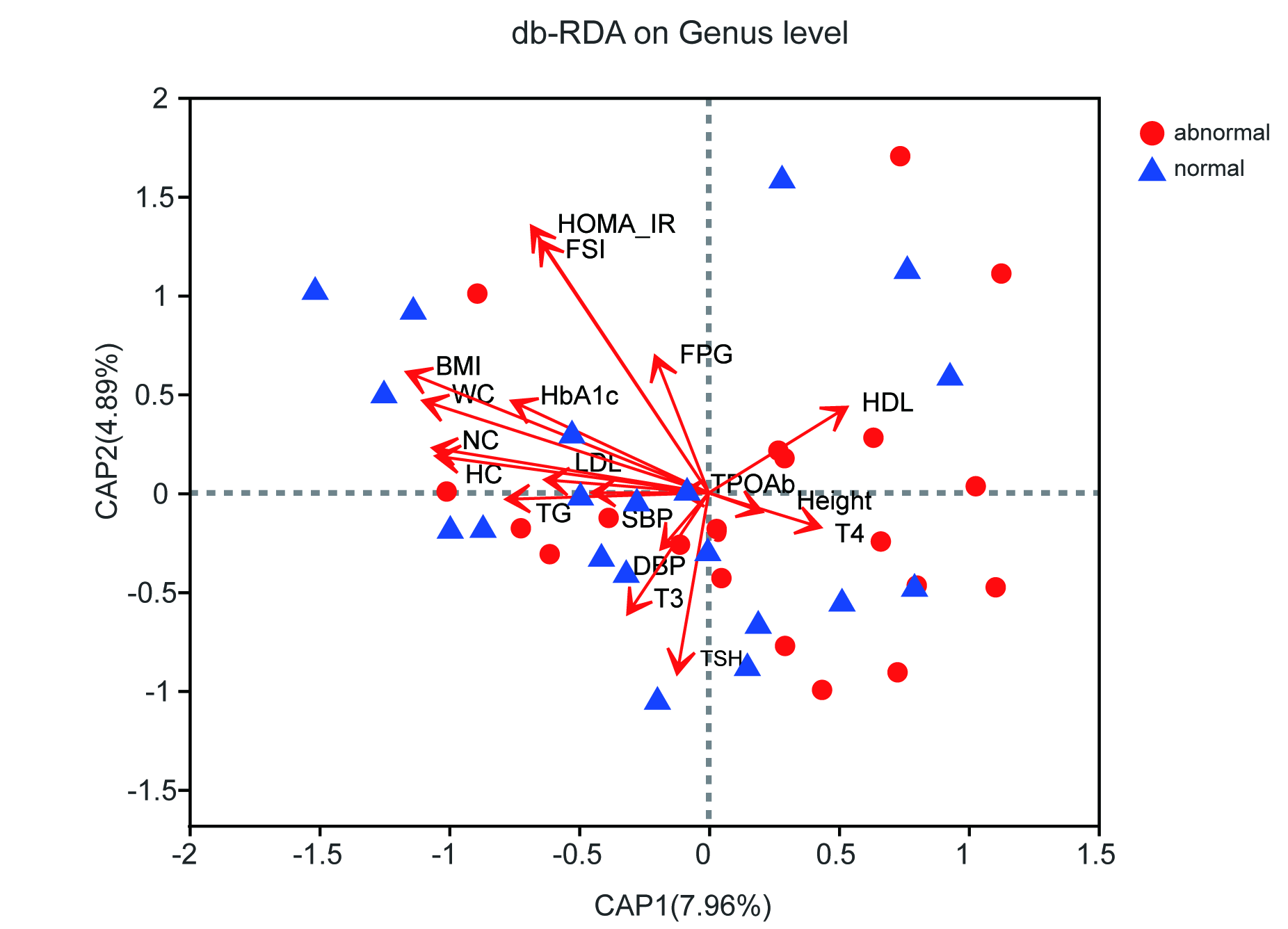

Supplement: Supplementary Figure 2 — Unweighted Unifrac db-RDA reflected the relationship between salivary microbiome and clinical variables. Red indicates samples from the Abnormal Group and blue indicates samples from the Normal Group. The red arrow indicates the quantitative clinical variables. The length of the arrow represents the degree of influence (interpretation) of the clinical variables on the species data; the angle between the arrows represents positive and negative correlation (acute angle: positive correlation; obtuse angle: negative correlation; right angle: no correlation). [file Image_2.tif]

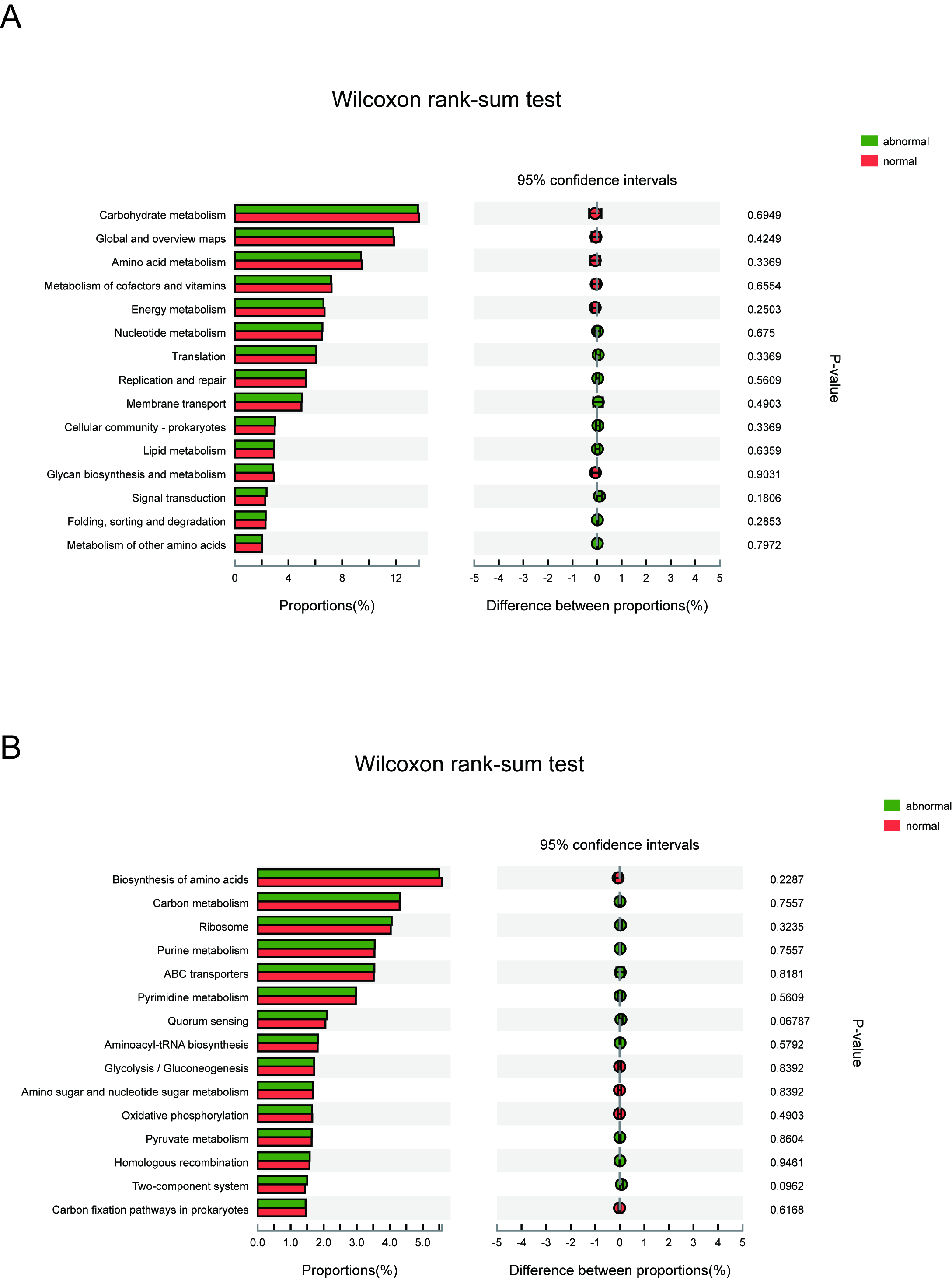

Supplement: Supplementary Figure 3 — Alterative predictive metagenome functional profiling along with serum TSH levels. (A) Relative abundance of predictive metagenome functional profiling of the top 10 abundant KEGG level 2 pathways (pathways with P(FDR)<0.05 are shown). (B) Relative abundance of predictive metagenome functional profiling of the top 10 abundant KEGG level 3 pathways (pathways with P(FDR)<0.05 are shown). [file Image_3.tif]
